# Predictors of mortality in ST-elevation MI patients: A prospective study

**DOI:** 10.1097/MD.0000000000010065

**Published:** 2018-03-02

**Authors:** Onur Zorbozan, Arif A. Cevik, Nurdan Acar, Engin Ozakin, Hamit Ozcelik, Alparslan Birdane, Fikri M. Abu-Zidan

**Affiliations:** a Department of Emergency Medicine, Eskisehir Osmangazi University, College of Medicine and Health Sciences, Eskisehir, Turkey; b Departments of Internal Medicine, College of Medicine and Health Sciences, United Arab Emirates University, Al Ain, United Arab Emirates; c Department of Cardiology, Eskisehir Osmangazi University, College of Medicine and Health Sciences, Eskisehir, Turkey; d Department of Surgery, College of Medicine and Health Sciences, United Arab Emirates University, Al Ain, United Arab Emirates.

**Keywords:** age, catheter activation time, modified shock index, mortality, Percutaneous Coronary Intervention, ST elevation myocardial infarction, systolic blood pressure

## Abstract

We aimed to define factors predicting mortality in patients having ST elevation myocardial infarction (STEMI) who had Primary Percutaneous Coronary Intervention (PCI) in our setting.

This is a prospective study on patients presenting to the emergency department with STEMI who underwent PCI during a 12-month period. Physiological parameters were calculated using the vital signs and age of patients. Time-based factors in the institutional protocol were collected. Univariate analysis was performed to define significant factors that affected mortality. Significant factors were then entered into a logistic regression model. Factors significantly affecting mortality were defined. Receiving operating characteristic curve was applied to define the best predictors of mortality.

A total of 167 consecutive patients were studied; 128 (76.6%) were males. The mean (SD) age of the patients was 61.9 (12.8) years. The logistic regression model showed that significant factors were age (*P* = .002), Modified Shock Index, MSI (*P* = .028), systolic blood pressure (*P* = .028), and time between consultation and activation of catheter laboratory (*P* = .047). The cut-off points with best prediction of mortality were age of 71.5 years, systolic blood pressure of less than 95 mmHg, MSI of 0.85, and a time more than 3.5 minutes between consultation and activation of catheter laboratory.

Our study shows that significant predictors of 30-days mortality of STEMI were age, systolic blood pressure on presentation, MSI, and the time between consultation and catheter laboratory activation. Improving prehospital resuscitation and activation of the catheter laboratory by emergency physicians may reduce mortality in our setting.

## Abstract 2

Supplemental Digital Content is available in the text

## 1 Introduction

There are 2 million patients with coronary heart disease in Turkey, with 160,000 new cases every year.^[1]^ Despite advancements in cardiac interventions, acute myocardial infarction is still one of the global leading causes of death.^[2]^ Its incidence is increasing.^[3]^ ST elevation myocardial infarction (STEMI) accounts for up to 40% of all acute coronary syndrome hospital admissions.^[4]^ One of the most important factors in treating STEMI is to achieve early reperfusion.^[2]^ Primary Percutaneous Coronary Intervention (PCI) is superior to fibrinolytic therapy, especially if it can be applied in less than 90 minutes.^[5]^ PCI time and other time frames before the PCI play an important role on mortality and morbidity, and may vary in different settings.

Nevertheless, mortality is affected by other factors including age,^[6]^ geographic region,^[7]^ gender,^[8]^ setting, time-based delays, and shock on presentation.^[9–11]^ Therefore, assessment of local protocols for quality improvement is necessary. Simple physiological parameters such as Shock Index (SI), and Modified Shock Index (MSI)^[12,13]^ were useful for predicting mortality of STEMI patients. There are other physiologic parameters, which were studied in critically ill patients including trauma and were shown to predict mortality.^[14,15]^ Nevertheless, this is not defined in STEMI patients. We aimed to define factors predicting mortality in patients having STEMI who had PCI in our setting.

## 2 Methods

### 2.1 Ethical approval

This study was reviewed and approved by the Research Ethics Committee of the College of Medicine of Eskisehir Osmangazi University (Reference No: 2011–291).

### 2.2 Study design and setting

This is a prospective study on patients presenting to the emergency department (ED) with STEMI who underwent PCI. The study was run during a 12-month period (November 1, 2011 – October 31, 2012). It was held at the Department of Emergency Medicine (EM) of Eskisehir Osmangazi University Medical Center. The department treats about 75,000 adult emergency patients every year.

### 2.3 Participants

For recruitment of patients, the following inclusion criteria were used: age 18 years or older, presentation with chest pain or chest pain equivalent symptoms, presence of ST elevation in 2 consecutive leads or new left bundle branch block in the initial ECG, the first STEMI, and performance of primary percutaneous coronary angioplasty. Exclusion criteria were: patients younger than 18 years old, suspicion for other reasons of ST elevation, patients who did not accept PCI, and patients who received thrombolytics. A written informed consent was taken from all patients before entering into the study.

### 2.4 Data collection

**Figure 1:**
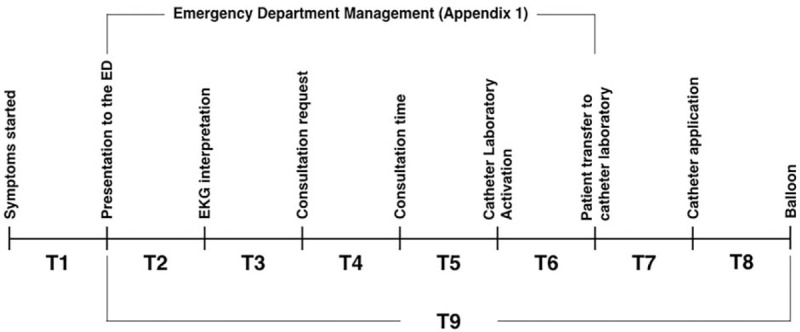
Time periods. T1: Duration of symptoms, T2: time between presentation and ECG interpretation, T3: time between ECG interpretation and consultation request, T4: time between consultation request and consultation, T5: time between consultation and catheter laboratory activation, T6: time between catheter laboratory activation and patient transfer to catheter laboratory, T7: time between patient transfer and catheter application, T8: time between catheter application and balloon, and T9: time between presentation to the ED and balloon.

A standard patient management protocol of our institution was used in this study (Appendix 1). A 1-hour presentation about the study protocol was given to nurses, residents, and faculty members of emergency and cardiology departments. The study forms were filled by the EM residents on their clinical shifts. Patient 12 leads ECGs were taken by Nihon Kohden Cardiofax GEM 9022 K with settings of 25 millimeters (mm) per second and 10 mm/millivolt calibrations. According to our institution protocol, all STEMI patients underwent a PCI. The PCI, using Philips Angodiagnost 5, was done by an interventional cardiologist. The PCI laboratory is located 30 meters away from the ED. Patients were then admitted to the coronary care unit (CCU). Mortality was followed for 30 days. Patient demographics, vital signs, presentation type (ambulance or by walking), chief complaint at the presentation (typical, atypical), and time related data were collected. Figure 1 shows the time line of the study and descriptions of the related data. Physiological predictors were calculated by using the vital signs and age of the patients (Appendix 2). Data were manually entered into an excel sheet by a senior EM resident. Data accuracy was audited by an EM residency core faculty member and a chief resident.

### 2.5 Statistical analysis

Patients were divided into 2 groups: those who died and those who survived. Univariate analysis was performed to define significant factors that affected mortality. Non parametric statistical methods were used to compare these 2 groups. Mann–Whitney *U* test was used for continuous or ordinal data and Fisher exact test for categorical data. We have used nonparametric statistical methods because the number of those who died was small (less than 20). These statistical methods are advised in this condition because they compare the ranks and a normal distribution is not needed.^[16]^ Significant factors were then entered into a backward logistic regression model to define factors significantly predicting mortality. Receiving operating characteristic (ROC) curve was applied to define the best cut off points for predicting mortality. Data were analyzed with PASW Statistics 21 (SPSS Inc; IBM SPSS Statistics for Windows, Version 21.0. Armonk, NY: IBM Corp.). For all analyses, a *P* < .05 was accepted to be significant.

## 3 Results

Three hundred fifty-seven patients with myocardial infarction were diagnosed during the study period. One hundred ninety-two patients were STEMI. One-hundred-sixty-seven consecutive patients who fulfilled inclusion criteria were enrolled into the study. One-hundred-twenty-eight (76.6%) were males. The mean (SD) age of the patients was 61.9 (12.8) years. The mortality was 9% (15 out of 167). Significant factors that affected mortality on univariate analysis of demographic and physiological parameters (Table 1) were age (*P* < .0001), blood pressure age index, BPAI (*P* < .0001), mean arterial pressure, MAP (*P* < .0001), Modified Shock Index, MSI (*P* = .028), Rate Over Pulse Pressure Evaluation Index, ROPE (*P* = .004), systolic blood pressure, SBP (*P* = .002), Shock Index, SI (*P* = .017), shock index age, SIA (*P* < .0001), and Thrombolysis in Myocardial Infarction Score, TIMI (*P* < .0001). Significant times affecting mortality are shown in Table 2. These included T1 (*P* = .017), T2 (*P* = .041), T3 (*P* = .008), T5 (*P* = .048), and T9 (*P* = .044).

**Table 1:**
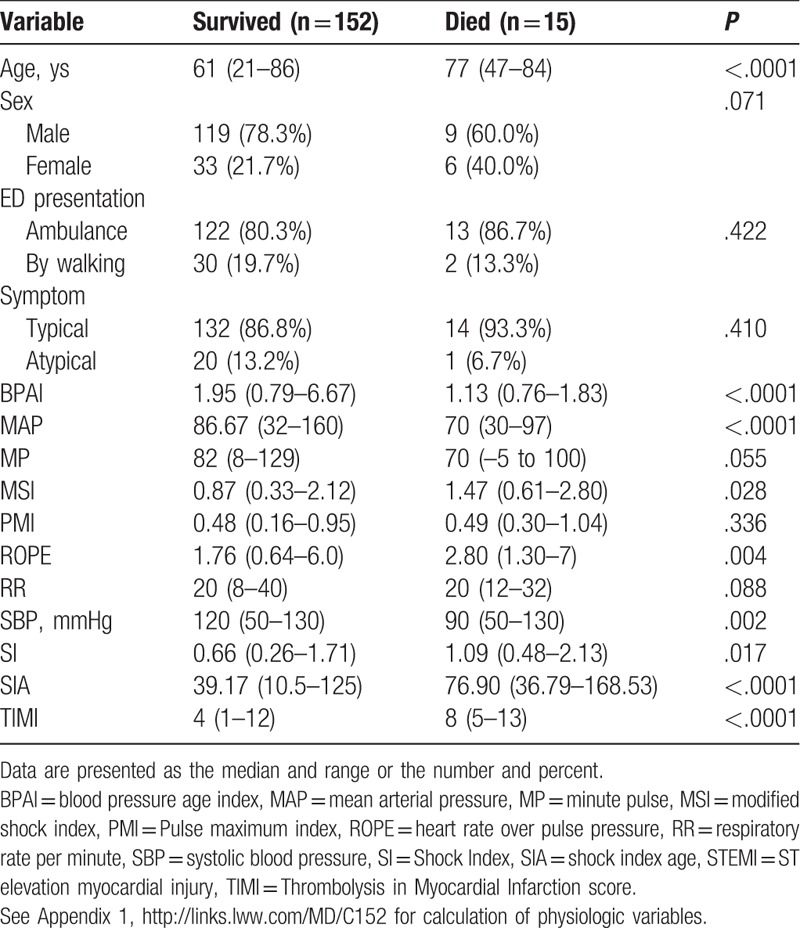
Univaraiate analysis comparing STEMI patients who survived and those who died.

**Table 2:**
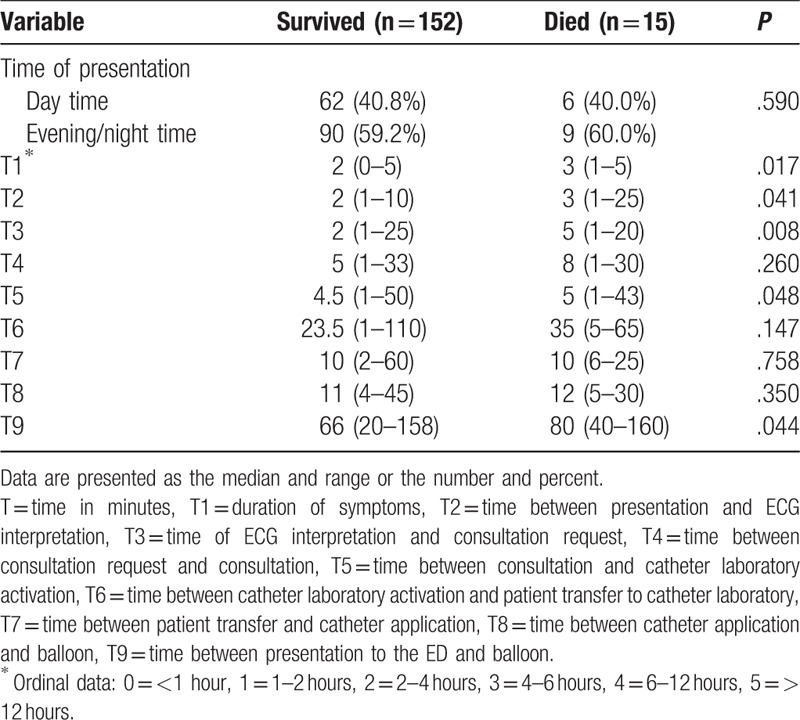
Univariate analysis of time dependable variables comparing STEMI patients who survived and those who died.

**Figure 2:**
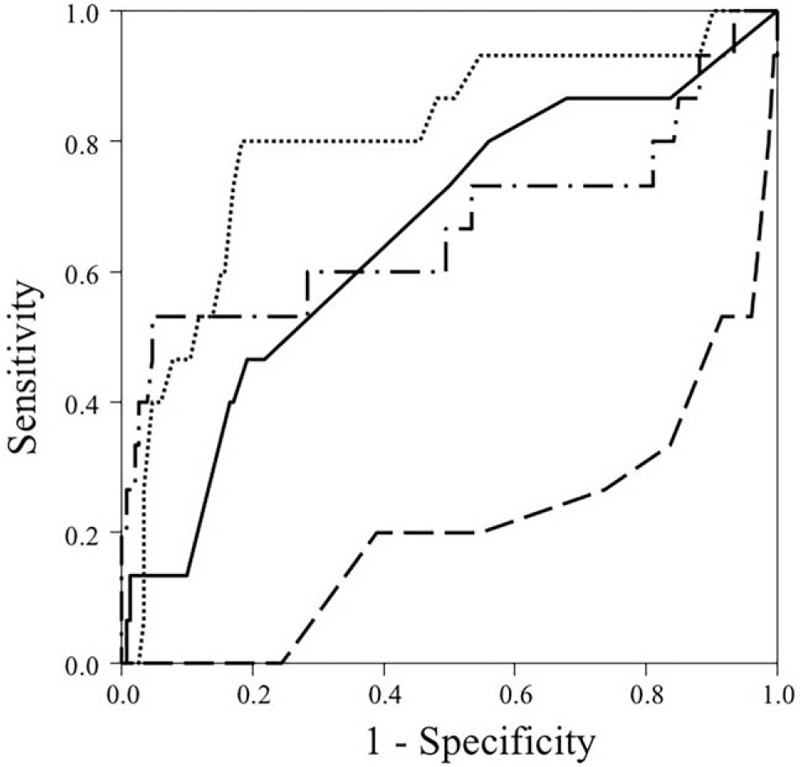
Receiver operating characteristic (ROC) curve for the best 4 variables that predicted mortality in the logistic regression model in 167 consecutive ST elevation myocardial injury patients. Age = dotted line, Modified Shock Index (MSI) = dashed dotted line, Consultation to Catheter Laboratory Activation Time (T5) = solid black line, Systolic Blood Pressure (SBP) = dashed line.

Backward logistic regression model defining significant predictors for mortality was highly significant (Nagelkerke R^2^: 0.51, *P* < .0001) (Table 3). The significant factors were age (*P* = .002), MSI (*P* = .028), SBP (*P* = .028), and the time between consultation and activation of catheter laboratory (*P* = .047). The areas under the curve of the significant variables are shown in the Figure 2. Best cut-off points for prediction and sensitivity and specificity of these points are shown in Table 4. Decrease in SBP, and increase in the other 3 factors caused an increased mortality.

**Table 3:**
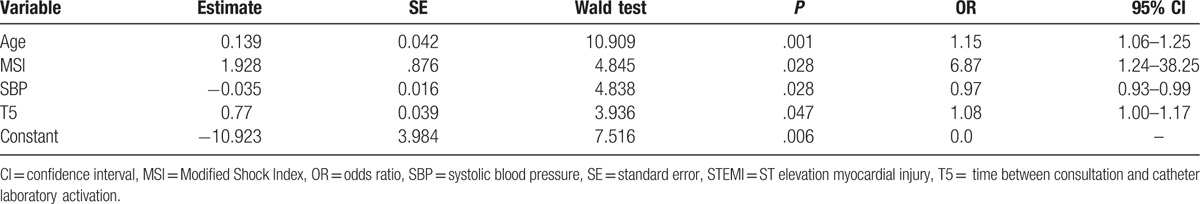
Backward logistic regression model defining significant predictors of mortality for patients with STEMI who had primary percutaneous coronary intervention (n = 167).

**Table 4:**
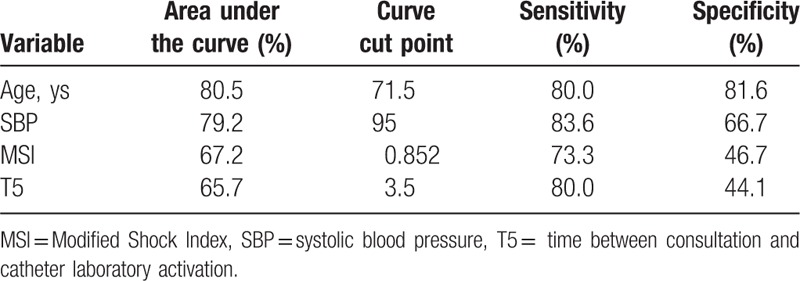
Area under the curve of significant predictors of mortality; the cut-off points; and their sensitivity and specificity.

## 4 Discussion

Defining mortality predictors of STEMI, which is affected by multiple factors, is important.^[6–11]^ We found that the most significant predictors of 30-day mortality for STEMI in our setting were age, MSI, SBP on presentation, and the time between consultation and catheter laboratory activation.

Although it was not significant, we found a trend in hospital mortality for the female gender similar to the results of De Luca et al.^[17]^ Patients who died were older than who survived. Age is a strong predictor of outcome in myocardial infarction and was recognized as 1 of the 5 prognostic factors in the GUSTO-1 study.^[18]^ Although TIMI risk score uses age above 70 years as a cut score, some studies showed that mortality is considerably higher at the age of 75 years and above.^[18,19]^ Spyridopoulos reported that age above 75 years has 3.5 times increased risk for mortality in patients with STEMI who had PCI.^[20]^ Similar to TIMI's age cut-off, we found that the highest sensitivity and specificity for predicting mortality to be at age 71.5 years. The odds of dying increased by 15% for each increased year of age.

Systolic blood pressure was lower in patients who died in our study. Similarly Gevaert et al^[21]^ showed that systolic blood pressure less than 100 mmHg increased mortality by 3.5 times. Our cut-off level which had the highest sensitivity and specificity for predicting mortality was a SBP of 95 mmHg. The odds of dying increases by 3% for each decrease of 1 mmHg. The physiological variables of our patients were recorded at presentation. Majority of our patients presenting with hypotension were brought to the ED by ambulances. Resuscitative efforts in the pre-hospital setting could have possibly improved the survival of our patients. MSI was shown as a strong predictor of ED patient mortality compared with heart rate and blood pressure.^[22]^ Similar to our results, Shangguan et al^[13]^ reported that patients with STEMI having a high MSI showed higher mortality. Although in their report, abnormal MSI was defined as ≥1.4, we found that the highest sensitivity and specificity cut-off point to be at 0.85 in our study.

Early PCI decreases mortality of STEMI patients. The time delay in PCI would increase the mortality.^[23–26]^ The American Heart Association and European Society of Cardiology recommended that the door to balloon time should be less than 90 minutes.^[27,28]^ We found longer door to balloon time (T9) in our patients who died. There are various potential time delays for the door to balloon time including ECG interpretation time,^[29]^ and the activation time of catheter laboratory.^[30]^ It should be acknowledged that each potential time can vary in different settings. Therefore, defining the exact delaying points may improve local protocols. We studied seven time periods (T2-8, Fig. 1). Although the time between presentation to interpretation of ECG, ECG interpretation and consultation request (T3), consultation and catheter laboratory activation (T5), and door to balloon (T9) were found significant in the univariate analysis, the backward logistic regression model defined that the time between consultation and catheter laboratory activation (T5) was the only significant factor predicting mortality. Every minute over 3.5 minutes increases the odds of dying by 8% in our setting. Immediate activation of catheter laboratory by emergency physicians after the STEMI diagnosis have achieved a decreased the median time of 27 to 38 minutes in door to balloon time.^[30–33]^ Modifying our institutional protocol to improve time periods in the management of STEMI patients undergoing PCI may decrease mortality.

We have to acknowledge that our study has certain limitations. This is a single center study. Because our institution is a tertiary care center, our data and results might have been affected due to a possibility of receiving more severe cases. Our city has another high-volume state hospital which has PCI capability. This may have attributed to the small sample of our study. However, to address that concern, we have used non-parametric methods which are advised for a small sample size. Nevertheless, there were highly significant findings indicating that the sample size was proper because the effect was large.

In summary, our study shows that significant predictors of 30-day mortality of STEMI were age, SBP on presentation, MSI, and the time between consultation and catheter laboratory activation. Improving prehospital resuscitation and activation of the catheter laboratory by emergency physicians may reduce mortality in our setting. A multi-centric study in our country is needed to address the role of prehospital care in stabilizing the vital signs, the role of emergency physicians in the sequence of the door to balloon time, and their effect on mortality.

## Supplementary Material

Supplemental Digital Content
