# Supplementary material for: Predictors of mortality in ST-elevation MI patients: A prospective study
Source: Medicine (Baltimore). 2018 Mar 2;97(9):e0065. doi: 10.1097/MD.0000000000010065 (PMC5851714; doi:10.1097/MD.0000000000010065)
Supplement: Supplemental Digital Content [file medi-97-e0065-s001.docx]

**Appendix 1:** The standard protocol in the Emergency Department

| - All patients with chest pain or chest pain equivalent symptoms were categorized as emergency by triage and located in the monitored bed area - Vitals were taken by nurse - Oxygen (4-6 L/min) was started if necessary (SatO2 < 94%) - IV line(s) inserted, preferably on antecubital area - Patient was evaluated by Emergency Medicine attending or senior resident. Simultaneously with the evaluation the aim was the completion of interpretation of the patient’s ECG less than 10 minutes. - Patient diagnosed with STEMI underwent cardiology consultation. STEMI was defined as ST-segment elevation ≥1 mm in 2 contiguous leads (or ≥2 mm in V1 to V3 leads) or new left bundle branch block together with chest pain for >30 minutes and/or evidence of myonecrosis with elevated troponin I. - 300 mg acetylsalicylic acid and 600 mg clopidogrel were given to patients appropriately. - Patient were given 0.4 mg nitroglycerine sublingually, with maximum repetition three times over 20 minutes. - IV nitroglycerine infusion was started to patients 10 microgram per minute till to the maximum dose of 100 micrograms per minute. - 2 mg IV Morphine by 5-15 minutes’ intervals was given to patients who has chest pain till the maximum 0.1 mg/kg dose. - IV or oral beta blocker was given to patients - Heparin was given in catheter laboratory as per protocol |
| --- |

STEMI: ST elevation myocardial injury. IV: intravenous, mg: milligram, kg: kilogram. PCI: primary percutaneous coronary intervention. All drugs were applied if there are no contraindications for patients. Heparin was given in catheter laboratory as per protocol. All patients underwent to PCI and admitted to coronary care unit after the procedure.

**Appendix 2**: Description and calculation of physiological predictors of mortality

| Parameter | Abbreviation | Formula |
| --- | --- | --- |
| Blood pressure age index [14] | BPAI | Systolic blood pressure / age |
| Mean Arterial Pressure | MAP | [(2 x DBP) + SBP]/3 |
| Minute pulse [14] | MP | Maximum heart rate - heart rate |
| Modified shock index [13] | MSI | Heart rate / mean arterial pressure |
| Pulse maximum index [14] | PMI | Heart rate / maximum heart rate |
| Rate over pulse pressure evaluation index [14] | ROPE | Heart rate / pulse pressure |
| Shock index [12] | SI | Heart rate / systolic blood pressure |
| Shock index age [14] | SIA | SI x patient’s age |

Maximum heart rate is calculated as 220 – Age. DBP: diastolic blood pressure. SBP: systolic blood pressure.
